# Descriptive study of the patients treated at the clinic “Integrated Dentistry for Patients with Special Needs” at Complutense University of Madrid (2003-2012)

**DOI:** 10.4317/medoral.20030

**Published:** 2015-02-07

**Authors:** Marta Monteserín-Matesanz, Germán C. Esparza-Gómez, Begoña García-Chías, Carmen Gasco-García, Rocío Cerero-Lapiedra

**Affiliations:** 1 Degree in Dentistry (UCM). MSc in Dental Sciences (UCM). Department of Medicine and Buccofacial Surgery, Faculty of Dentistry, Universidad Complutense de Madrid, Spain; 2 Professor. Department of Medicine and Buccofacial Surgery, Faculty of Dentistry, Universidad Complutense de Madrid, Spain; 3 Professor. Department of Pharmacology. Faculty of Medicine, Universidad Complutense de Madrid, Spain

## Abstract

Objectives: To study clinical and epidemiological characteristics of the patients treated at the clinic “Integrated Dentistry for Patients with Special Needs (Special Care Dentistry)” at Complutense University of Madrid (UCM), as well as to know the dental treatments performed in these patients and the modifications from the usual treatment protocol. The information obtained from the results could also be applied in order to assess the needs of dental students education about this type of patients. Study Design: Medical records review of all the patients referred to the clinic of “Integrated Dentistry for Patients with Special Needs”, performing a retrospective cross-sectional study analyzing their main pathology, ASA risk score (Classification system used by the American Society of Anesthesiologists to estimate the risk posed by the anesthesia for various patient conditions), pharmacological treatment, what kind of dental treatment was necessary, whether the patient was treated or not, and if it was required to change any procedure due to the patient health status (sedation or antibiotic prophylaxis). Results: The number of patients referred to the clinic was 447, of whom 426 were included in this study. Out of them, 52,35 % were men and 47,89 were women, with a mean age of 49,20 years. More frequent pathologies were cardiovascular or cerebrovascular diseases (24,29 %), infectious diseases (12,41%), endocrine diseases (11,66%) and intellectual disability (8,85%). 70’18% of the patients were treated, with sedation being necessary in 9,03% of the cases and antibiotic prophylaxis in 11,70%. Conclusions: Given the high number of patients with some kind of pathology and the amount of medicines that they use, it seems necessary for dentistry students to have a specific training regarding how to handle and treat these patients, so they will be able to provide them the best possible care.

** Key words:**Patients with special needs, medically compromised patients, dental treatment, special care dentistry.

## Introduction

Over the last few years, research and progress of medical science have allowed an important development in the treatment of patients with systemic diseases, and have contributed to an improvement not only in life expectancy but also in its quality. It’s becoming more common the demand of dental treatment by patients with diverse systemic diseases, elderly patients with several pathologies, patients with physical or mental disabilities, immunocompromised patients and cancer patients.

Many of the patients attending dental clinic suffer some kind of systemic diseases, acute or chronic, that require an accurate knowledge of the pathology, as well as their implications and interactions with dental treatments. The dentist needs to be familiar with cardiovascular, respiratory, immune, endocrine and metabolic diseases in order to be able to treat these patients correctly. Immunocompromised patients, patients with cancer, patients taking anticoagulant drugs, patients with HIV or hepatitis may require specific dental treatment. Physical and intellectual disabled are challenging for the dentist, as they could need changes from the usual treatment protocol. All of these are considered “patients or individuals with special needs” although this denomination might not be the most appropriate, as it can create some kind of stigma (1).

In Dentistry, the term “patient with special needs” includes not only both adults and children who are under medication due to their systemic disease and the disabled patients who have difficulties managing behavior or motor skills, but also patients with pathology in the oral cavity that makes dental treatment complicated. Therefore, the term “patients with special needs” include all those patients, whose medical, physical or social situation make it necessary to consider a wide range of assessment and care options in order to provide dental treatment. These individuals include, but are not limited to people with developmental disabilities, cognitive impairment, complex medical problems, significant physical limitations and the vulnerable elderly (2,3).

## Patient and Method

All the patients referred to the clinic “Integrated Dentistry for Patients with Special Needs (Special Care Dentistry)” at Dentistry Faculty at UCM between academic year 2003/2004 and 2011/2012 were included in the study. The information was collected from the patient’s medical records, which was obtained both manually, from the General Archives from the Dentistry Faculty (UCM), and by computer search in “Salud Dental Suite Program®”, version 1.16.0 (Two-Ten Health, Dublin, Ireland).

Willing to facilitate the data analysis, the information from medical records was divided in different categories.

- Main pathology: a patient can have more than one disease.

- ASA risk score (Classification system used by the American Society of Anesthesiologists to estimate the risk posed by the anesthesia for various patient conditions).

- Pharmacological treatment.

- Dental treatment at the clinic and if sedation or antibiotic prophylaxis (to prevent local infection or as prophylaxis for infective endocarditis) was necessary.

- Type of dental treatment performed: the same patient could have different kind of treatments.

The analysis was carried out with Microsoft Excel 2010 for Windows XP.

## Results

The number of patients referred to the clinic between academic years 2003/2004 and 2011/2012 was 447. Medical records from 365 of the patients were obtained after manual search, and 82 were obtained through “Programa Salud”®. All the medical records were analyzed in a descriptive cross-over retrospective study.

From 447 patients, 21 were excluded, due to their lack of pathology making them susceptible for being treated in this clinic. Thus, the number of patients included in the study was 426. Out of them, 223 were men (52,35 %) and 204 were women (47,89%). The mean age was 49,20 years, with a standard deviation of 16,48 (range 14-86). Among men, the mean age was 49,16 (SD= 16,47; range 14-85) and among women it was 49,21 (SD=16,49; range 14-86).

Main pathology data from the patients are included in  Table 1. The most common pathology was cardiovascular and cerebrovascular disease (24,94%) ( Table 2), followed by infectious diseases (12,41%) ( Table 3), endocrine pathology (11,66%) and intellectual disability (8,85%).

**Table 1:**
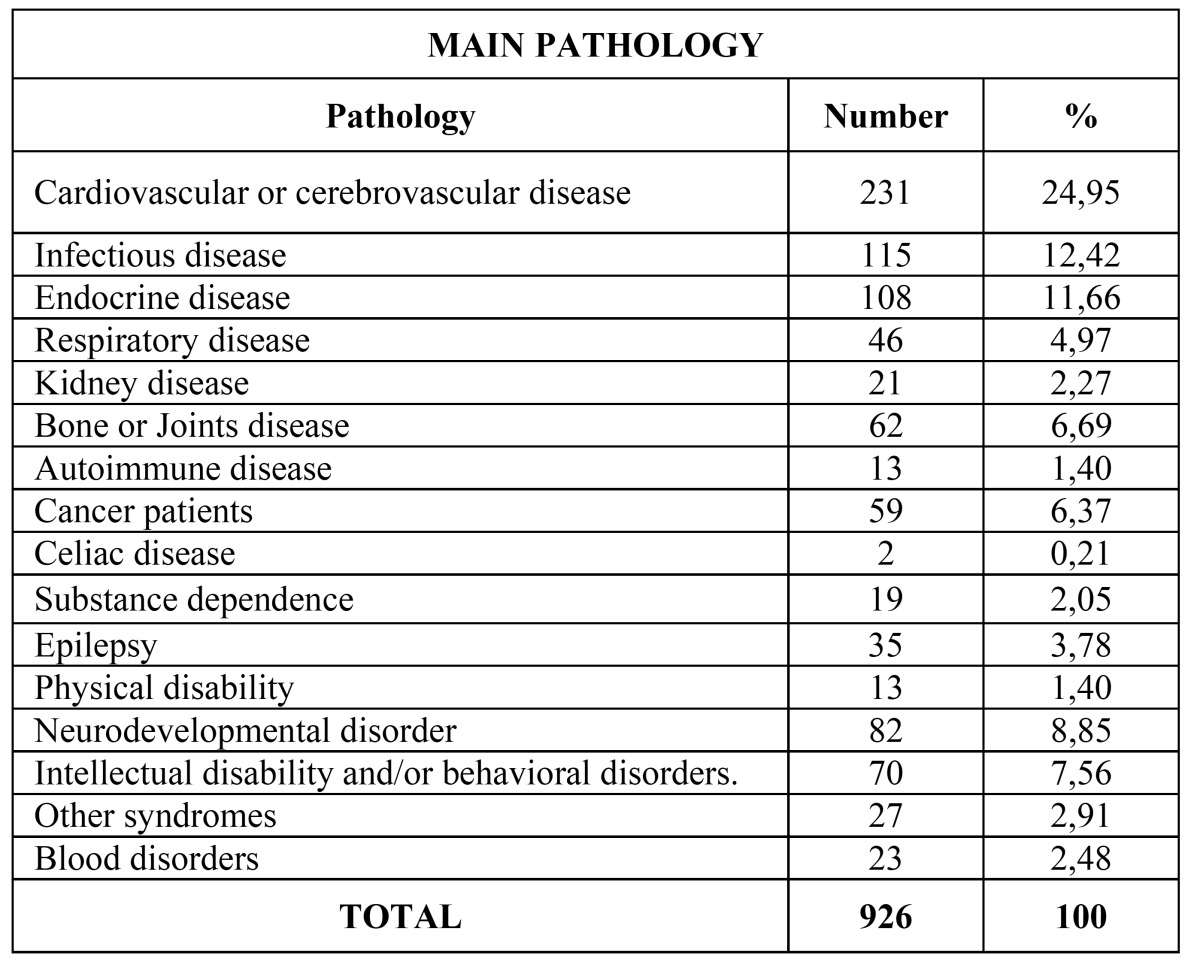
Percentage distribution of the main pathology of the patients included in the study.

**Table 2:**
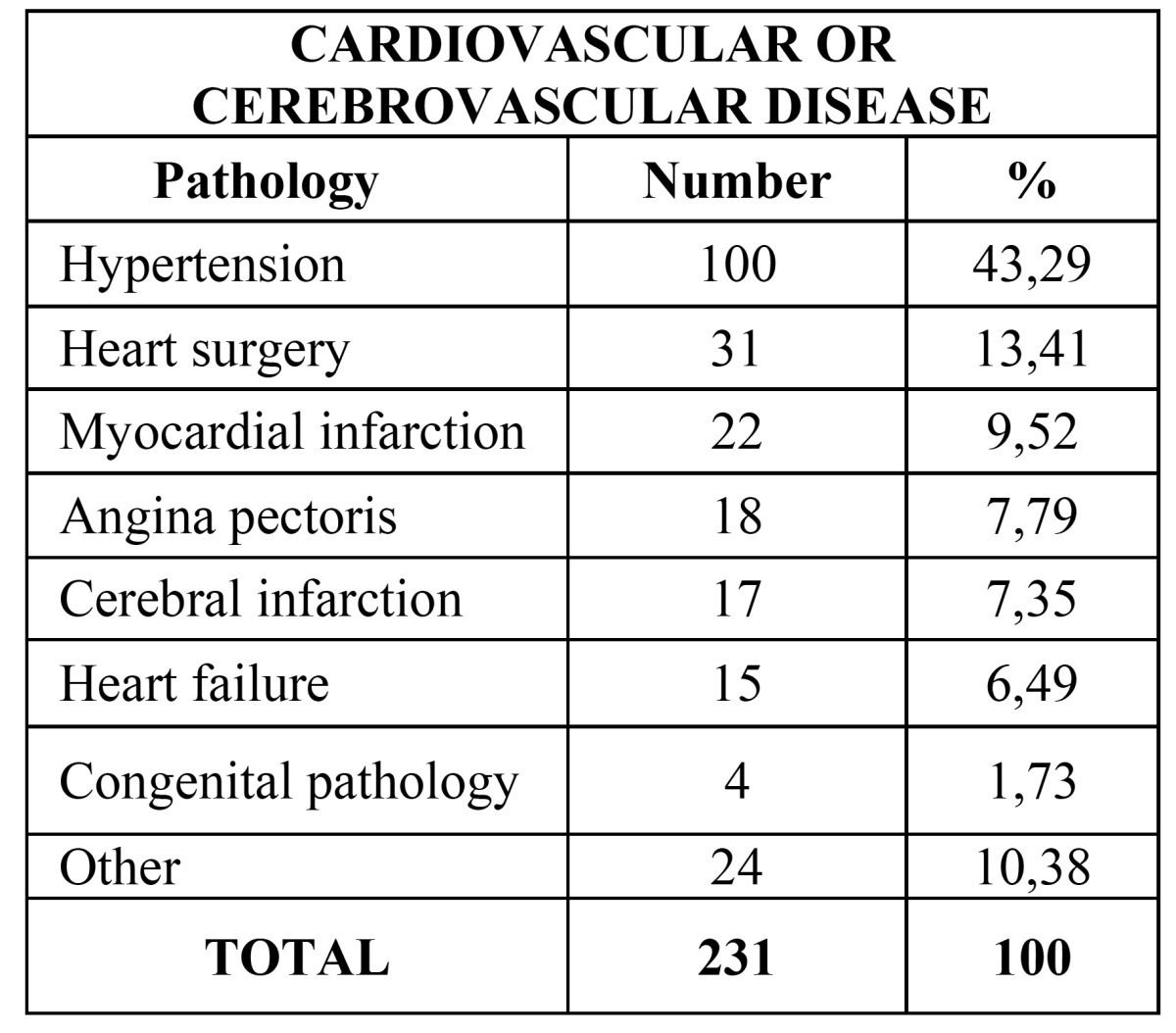
Percentages distribution of cardiovascular and cerebrovascular pathology.

**Table 3:**
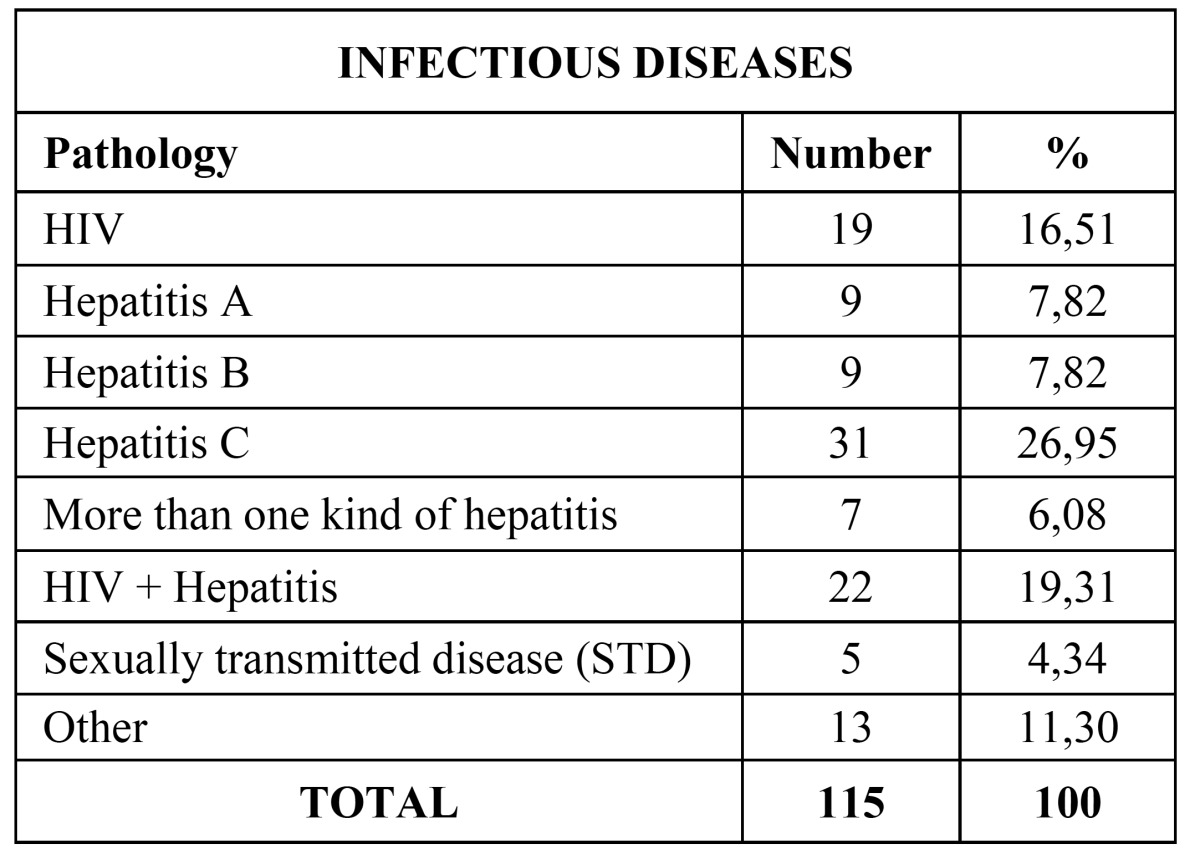
Percentage distribution of infectious diseases.

About ASA risk score, 99 of the patients were classified as ASA I (23,23% - Group ASA I included patients without anesthetic risk but with behavioral, comprehension or mobility disorders whose situation advised referral to our clinic. Their oral process for intervention were located, without producing systemic alteration), 166 as ASA II (38,96%), 125 ASA III (29,34%) and 36 ASA IV (8,45%). None of the patients was classified as ASA V.

Regarding pharmacological treatment, 14,28% of our patients were not under any medication when they received dental treatment, 83,85% were being treated pharmacologically and in 2,34% of the cases there was lack of data concerning drug intake. Among those who had some kind of drug treatment, 60,95% were using between 1 and 4 different medicines, 30,89% were on treatment with 5 to 8 different drugs and 8,14% were being treated with more than 8 medicines. We also assessed what kind of medicines were consuming our patients, with special attention to those that could somehow interfere with dental treatment. 23,59% of our patients who were under some medication were being treated with anti platelet or anticoagulant drugs, 12,35% of our patients were insulin-dependent diabetics or were being treated with oral anti diabetics, and 3,93% of the patients were under treatment with biphosphonates. All the other patients under pharmacological treatment (60,11%) were using drugs different from those in these groups.

Dental treatment was performed in 70,18% of the 426 patients included in the study, versus 29,81% who were not treated. Causes for not being treated were diverse: patient’s resignation (24,24%), not attending the appointments (22,83%), patient referral to another Faculty Department (17,32%), not being able to contact the patient (14,17%), patient being treated somewhere else (9,44%), treatment was not necessary (4,72%) and another causes (7,08%).

Among the patients who were treated, we evaluated the need of sedation and or antibiotic prophylaxis. Of the 299 patients treated, 9,03% required sedation. It was performed once in 44,44 % of the patients, twice in 25,92%, three times in 14,81% and four or more times in 14,81%. Concerning antibiotic prophylaxis prior to dental treatment, it was necessary in 11,70% of the cases. Out of these, 57,14% needed it as prevention for infective endocarditis and 42,85% to prevent local infection.

Dental treatments performed in our patients are described in  Table 4. Most common dental treatment were conservative dentistry treatments (90,30%), periodontal treatments (76,25%) and surgical treatments (46,48%). Prosthetic treatments were necessary in 26,08% of the patients and mouth guards in 5,35%.

**Table 4:**
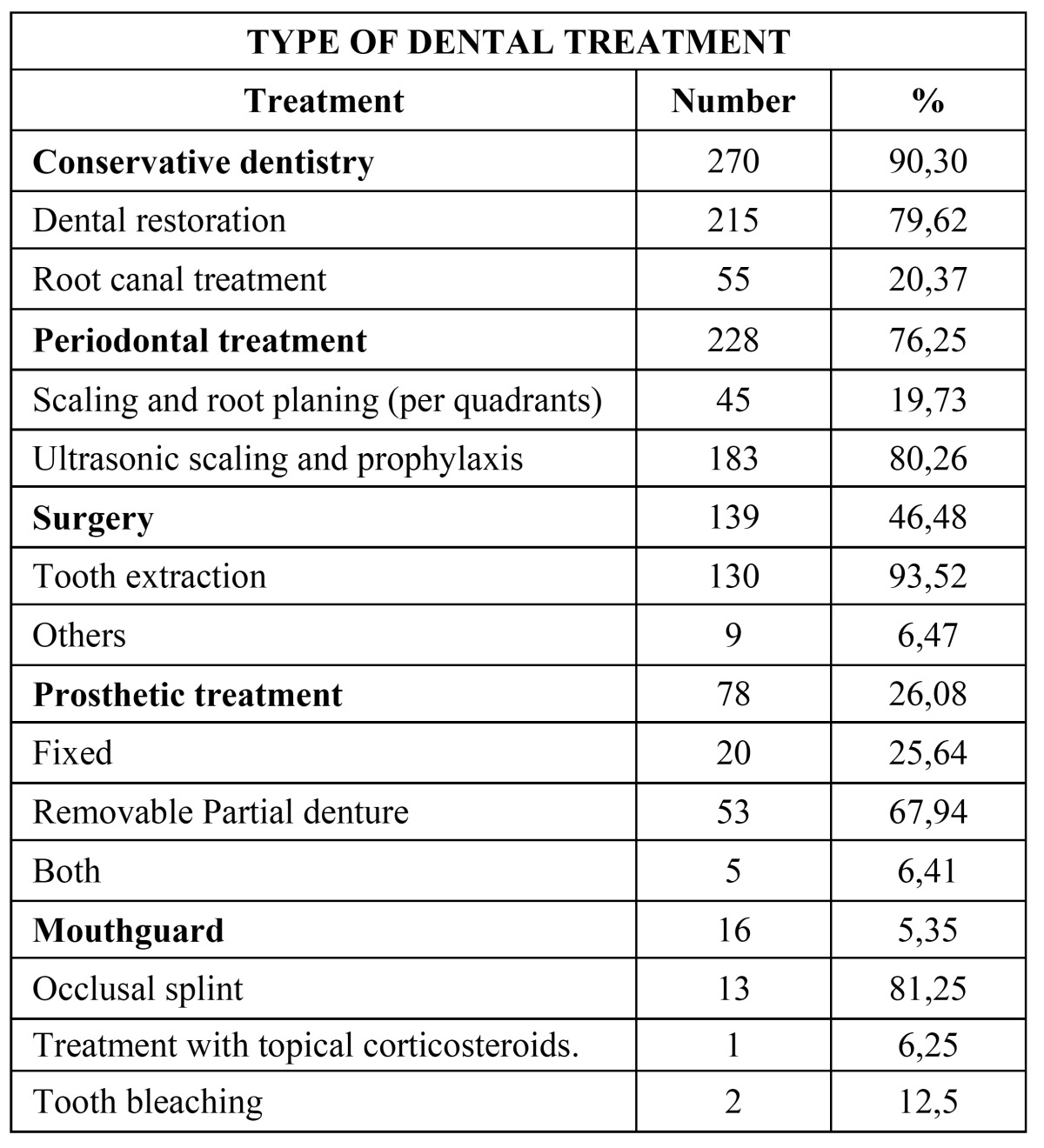
Percentage distribution of the treatments performed in the patients included in the study.

## Discussion

Descriptive studies are essential to establish treatment protocols and to understand treatment needs of a population. In Spain, as far as we know, there are not previous studies analyzing epidemiological data regarding patients with special needs conducted in Dentistry Faculties. Being this one the first study on the topic, it makes it difficult to compare with similar research.

In our research, we included 426 medical records from patients treated in the “Integrated Dentistry for Patients with Special Needs”, 223 men and 204 women. Most of these patients had, at least, one systemic pathology, with coexistence of two or more pathologies in some of the patients, being this the reason for their referral to our clinic.

Cardiovascular and/or cerebrovascular diseases were the most common (24,94%), as reported on previous studies (4-9). Among the different pathologies included in this group, the most frequent was arterial hypertension (HT), which appeared in 43,29% of these patients. HT was also the most frequent disease in previous reports, as in Al-Bayaty *et al*. (6) with 12,6% of the patients suffering it, and Chandler-Gutierrez *et al*. (7), with 13,8%. Other studies, as Amado-Cuesta *et al*. (10) and Martinez-González *et al*. (8) also considered the HT as the most frequent pathology (44,9% and 40,5% respectively), although only patients over 65 were included. Our results are also compatible with the Spanish National Health Survey from 2011-2012 and also in the previous one from 2006, which stated that HT was the most prevalent chronic or long-standing problem or disease diagnosed by doctors in individuals over 15 years in Spain (11,12). However, it is important to note that not all the patients going to the dental clinic have the hypertension diagnosed and/or controlled, as revealed by Fernández-Feijoo *et al*. in their study from 2010 (13). Their results showed the importance of the dentist’s role in detecting undiagnosed hypertension. The health status of individuals requesting dental care is most commonly evaluated throughout the use of self-administered questionnaires. Nevertheless, these questionnaires have some limitations, as they require the patient’s collaboration, must be drawn up in a language that the patient understand, and they require confirmation of the replies by the dentist (4,14,15).

The second most common group of pathologies were infectious diseases (12,41%). In this group, the most prevalent disease was hepatitis C (29,95%), followed by coinfection by HIV and some type of hepatitis (19,13%) and infection by HIV (16,51%). It draws attention the high number of patients infected by hepatitis C virus and/or HIV in our study versus those found by Chandler-Gutierrez *et al*. (7) at theirs. The reason could be that we only analyzed those patients that had been referred to the “Integrated Dentistry for Patients with Special Needs” unlike them who included all the patients that attended to their University Dental Unit in approximately 6 months. Infectious diseases have great importance both for the dentist and the patient due to the risk of disease transmission, and thus precaution measures should be increased (16).

It is also important to take into consideration drug addict patients, who comprise a high risk group and with whom similar precautions to those for patients with infectious diseases should be taken (7). Chandler-Gutierrez *et al*. (7) reported a rate of 0,83% of patients taking drugs while we documented 2,05% of our patients as drug depend ants.

The patients with some kind of endocrine pathology summed up a total of 11,66%. Out of them, 65,74% had diabetes, 25,92% hypothyroidism, 3,70% hyperthyroidism and the other 4,62% suffered from other type of endocrine disease. Similar to our study, other reviews report diabetes as the most common pathology among endocrine diseases (7,8,10). About 2% of the patients attending dental clinics suffer from diabetes, with about half of them unaware of their condition. Because of this, it is extremely important for the dentist to make sure the medical record reported by the patient is accurate, and to refer him or her to the doctor if there is any doubt (7). Nevertheless, well-controlled diabetic patients do not have a higher incidence of infections than the general population (10).

We consider of special interest the analysis of mentally disabled patients referred to our clinic, as they can be susceptible of needing sedation or other special measures for their treatment (17,18). It is estimated that about 15% of Spanish population suffer from some kind of disability, with moderate or severe impairment in 4-5% of the cases (17). Out of 426 patients included in our study, 8,85% suffered some kind of mental disability. We deem that patients with severe handicap who need dental treatment under sedation or general anesthesia should be referred to specialized professionals, so they can evaluate their dental status and their risk of suffering some complication and determine if it is convenient to perform the dental treatment or if the risk is not justified (17,18).

23,23% of the patients included in our study can be classified as ASA I, 38,96% as ASA II, 29,34% as ASA III and 8,45% as ASA IV. Previous studies by Chandler-Gutierrez *et al*. (7) and Al-Bayati *et al*. (6) categorize most of their patients as ASA I, with small percentages of patients ASA II, III or IV. These results contrast with ours, with most of our patients being included in ASA II and ASA III groups.

Due to the high prevalence of patients with systemic diseases, we consider that it is compulsory to record a medical history. Using a questionnaire can be useful in order to quickly classify a patient with the ASA risk score (7). Thereby, possible complications when carrying out the dental treatment can be avoided.

About 75% of patients over 55 are taking some kind of drug that helps to maintain vital functions (7,19). Carter *et al*. (20) confirm the existence of a progressive trend towards polypharmacy, being more common in patients over 65. Any medication consumption has side effects, and the more drugs a patient consume the risk of interactions with other medication prescribed by the dentist increase. Hence, it is of extremely importance to know and understand the patient’s medication (7,19). In our study, 14,28% of the patients would not be taking any kind of medicine, versus 83,56% who were under medication. Out of these, 60,95% were using between 1 and 4 different medicines, 30,89% were on treatment with 5 to 8 different drugs and 8,14% were being treated with more than 8 medicines.

Dental treatment was carried out in 299 patients (70,18%). It could seem a small number for nine academic years, but it is important to point out that the clinic was only operating during one semester, 3 hours per week. Furthermore, most of the patients were treated during several appointments in the same semester, so they could have the treatment plan completed, and also that most of the patients would be revised in successive years. Every year there would be new patients, but also all previous patients were revised, mostly in order to check oral hygiene and the implications of the lack of it.

It was necessary to perform moderate sedation in 27 patients (9,03%). Sedation is indicated in dentistry mostly in no cooperative patients, small children, medically compromised patients and patients with physical or mental disabilities, due to the difficulty of treating these patients in the dental chair (17,18). In our case, all of the patients that were treated under sedation had different disabilities, such as mental disability, cerebral palsy or Down syndrome. In most of the cases sedation was performd in order to be able to carry out all the dental treatment needed in just one session. Actually, the number of patients that were treated under sedation was relatively small, and in our opinion most of them could have been treated in any regular dental clinic.

After evaluating and analyzing all these data, we believe it is necessary to raise the idea of dental students receiving specific education about special care dentistry. Previous research have shown that not every dentist feel ready or is willing to treat this kind of patients (21-23). One reason of the dentist reluctance could be based on their education (2). Waldman *et al*. (22) determined back in 2002 that dental student consider that they do not receive enough theoretical and clinical training about the dental care that patients with special needs need. In 2005, Dao *et al*. (21) showed that dentists who felt well prepared were more likely to provide services for these patients and had a more positive attitude towards treating them. Chavez *et al*. (24) reported in 2011 that those dentists that have been prepared to treat patients with special needs during their education are more prone to treat them after university. There are some other additional factors that influence whether the dentist would treat or not these patients, such as the low compensation rates combined with the complex management issues and additional time and staffing that are required in special care dentistry. However, the role of the dental education received is crucial (3,21,25). Taking into consideration that most of the patients with mild or moderate health problems could be treated by general dentists, it is important to develop and implement more educational programs to train providers with the specialized skills required in special care dentistry (25).

Research has shown that those dentist who have received specific training, both theoretical and clinical practice on the treatment of patients with special needs, feel more comfortable with the idea of performing any treatment on these patients, and thus are more like to provide such care (2,21). Nonetheless several reports have demonstrated that most of the dentists feel that they have not received an adequate training. Cassamassimo *et al*. (26) informed in 2006 that only one out of four dentist had been educated in special care dentistry. As well as Dao *et al*. (21), these authors also found that the dentist who had not been exposed to these issues in lectures and clinical settings, were less likely to treat patients with special care needs (26). In addition, Wolff *et al*. (27) found in 2004 that 50% of dental students reported they had not received any clinical training for the management of patients with mental retardation, and that 75% said they only had little or no education or clinical training at all in special care dentistry. In 2006, Chmar *el al*. (28) published that only 6,2% of the students that had just graduated considered that they were well prepared to treat individuals with some kind of disability.

Given the high percentage of children and adults with disabilities and special needs, taking into account their substantial need for access to dental care, and considering that life expectancy of the population is greater each time, it seems crucial to prepare all future dentist in such way, so they are able to assess the treatment need for these patients and to provide basic care (29). It should be considered not to include in the group of patients with special needs those with multiple pathologies or under poly pharmacy, but limiting it to those who need to be treated under sedation or general anesthesia.

With all these things under consideration, we can conclude that much remains to be done in preparing general dentist in special care dentistry (30).
